# Effect of Sublethal Prenatal Endotoxaemia on Murine Placental Transport Systems and Lipid Homeostasis

**DOI:** 10.3389/fmicb.2021.706499

**Published:** 2021-07-30

**Authors:** Mila W. Reginatto, Klaus Novaes Fontes, Victoria R. S. Monteiro, Natalia L. Silva, Cherley Borba Vieira Andrade, Hanailly Ribeiro Gomes, Guinever E. Imperio, Flavia Fonseca Bloise, George Eduardo Gabriel Kluck, Georgia Correa Atella, Stephen G. Matthews, Enrrico Bloise, Tania M. Ortiga-Carvalho

**Affiliations:** ^1^Laboratory of Translational Endocrinology, Institute of Biophysics Carlos Chagas Filho, Universidade Federal do Rio de Janeiro, Rio de Janeiro, Brazil; ^2^Department of Physiology, Faculty of Medicine, University of Toronto, Toronto, ON, Canada; ^3^Lunenfeld-Tanenbaum Research Institute of Medical, Sinai Health System, Toronto, ON, Canada; ^4^Laboratory of Lipids and Lipoproteins Biochemistry, Institute of Medical Biochemistry Leopoldo de Meis, Universidade Federal do Rio de Janeiro, Rio de Janeiro, Brazil; ^5^Department of Obstetrics and Gynecology, Faculty of Medicine, University of Toronto, Toronto, ON, Canada; ^6^Department of Medicine, Faculty of Medicine, University of Toronto, Toronto, ON, Canada; ^7^Department of Morphology, Universidade Federal de Minas Gerais, Belo Horizonte, Brazil

**Keywords:** lipopolysaccharide, ATP binding cassette transporters, P-glycoprotein, breast cancer resistance protein, cholesterol, ABC sub-family G member 1, fatty acid binding protein associated with plasma membrane, *FAtty acid translocase*

## Abstract

Infection alters the expression of transporters that mediate the placental exchange of xenobiotics, lipids and cytokines. We hypothesized that lipopolysaccharide (LPS) modifies the expression of placental transport systems and lipid homeostasis. LPS (150 μg/kg; i.p.) treatments were administered for 4 h or 24 h, animals were euthanized at gestational days (GD) 15.5 or 18.5, and maternal blood, fetuses and placentae were collected. Increased rates of fetal demise were observed at GD15.5 following LPS treatment, whereas at GD18.5, high rates of early labour occurred and were associated with distinct proinflammatory responses. Lipopolysaccharide did not alter ATP-binding cassette (ABC) transporter mRNA expression but decreased fatty acid binding protein associated with plasma membrane (*Fabppm*) at GD15.5 (LPS-4 h) and increased fatty acid translocase (*Fat/Cd36*) mRNA at GD18.5 (LPS-4 h). At the protein level, breast cancer-related protein (Bcrp) and ABC sub-family G member 1 (Abcg1) levels were decreased in the placental labyrinth zone (Lz) at GD15.5, whereas P-glycoprotein (P-gp) and Bcrp Lz-immunostaining was decreased at GD18.5. In the placental junctional zone (Jz), P-gp, Bcrp and Abcg1 levels were higher at GD18.5. Specific maternal plasma and placental changes in triacylglycerol, free fatty acid, cholesterol, cholesterol ester and monoacylglycerol levels were detected in a gestational age-dependent manner. In conclusion, LPS-increased risk of fetal death and early labour were associated with altered placental ABC and lipid transporter expression and deranged maternal plasma and placental lipid homeostasis. These changes may potentially modify fetal xenobiotic exposure and placental lipid exchange in cases of bacterial infection.

## Introduction

Global estimates indicate that more than 15 million babies are born preterm every year (Harrison and Goldenberg, 2016). Preterm birth (PTB) occurs at higher rates in low- and middle-income countries and may range from 5 to 18% of all pregnancies worldwide (Goldenberg et al., 2009). Of particular importance, low-income countries have a higher incidence of histological chorioamnionitis (HCA)-related genitourinary (retrograde or ascending bacterial) and malarial (haematogenous) infections. These infections may lead to severe systemic and placental inflammatory response (Bloise et al., 2010; Novembri et al., 2011; Fontes et al., 2019) and become important triggers of inflammatory PTB pathways (Challis et al., 2009; Conti et al., 2015; Harrison and Goldenberg, 2016). Other routes/risk factors for infection-associated PTB include maternal periodontal disease, transplacental transfer of pathogens, iatrogenic infection from complicating amniocentesis or chorionic villous sampling (Conti et al., 2015; Nadeau et al., 2016).

The most common microbes observed in HCA are the gram-negative bacteria *Ureaplasma urealyticum, Mycoplasma hominis*, and *Escherichia coli* (Conti et al., 2015). Lipopolysaccharide (LPS), an endotoxin enriched in the cell wall of gram-negative bacteria, binds Toll-like receptor 4 (Tlr-4) and is widely used to model gram-negative bacterial infections (Beutler et al., 2003). Studies in mice have shown that LPS exposure alters the fetal-placental unit in a gestational age-dependent manner. In early pregnancy, it induces embryonic loss (Ogando et al., 2003) or miscarriage (Leazer et al., 2002), whereas in mid-pregnancy, it causes fetal death, fetal growth restriction (Guo et al., 2013) and fetal brain injury (Elovitz et al., 2011).

Infection and inflammation have the potential to disrupt the syncytiotrophoblast barrier by modulating the expression and function of ATP-binding cassette (ABC) transporters (Lye et al., 2015; Bloise et al., 2016; Do Imperio et al., 2018). These proteins are active transmembrane efflux transport systems that control the biodistribution of clinically relevant endogenous and exogenous substrates across the maternal-fetal interface. Examples of endogenous substrates include nutrients (cholesterol and other lipids), metabolites (bilirubin- and bile salt-conjugated compounds and oxysterols), steroid hormones (glucocorticoids, mineralocorticoids, oestrogens, progestogens, and androgens) and immunological factors (cytokines and chemokines). Examples of exogenous substrates include therapeutic drugs (antibiotics, antiretrovirals, synthetic glucocorticoids and NSAIDs) and environmental toxins (organochlorine and organophosphorus pesticides, ivermectin and bisphenol A). As a result, ABC transporters limit the transfer of potentially harmful substrates to the foetus and control transplacental passage of nutrients (mostly lipids) and other maternally derived substances in a gestational age-dependent manner (Imperio et al., 2018). In addition, the biodistribution of cytokines and chemokines within gestational tissues is modulated by the actions of placental ABC transporters and, as such, may be involved in the pathogenesis of PTB.

Importantly, cultured human primary villous trophoblast cells exposed to LPS and to the viral double-stranded RNA analogue polyinosinic:polycytidylic acid (polyI:C) exhibit markers of insulin resistance and increased amino acid uptake (Liong and Lappas, 2017). Treatment of trophoblast cells with cytokines, such as interleukin (IL)-1β and IL-6, elicited similar responses (Jones et al., 2009; Aye et al., 2013) indicating that infection may alter placental nutrient uptake and fetal transfer. However, the effect of infection on placental lipid uptake and fetal transfer is less well understood.

In the present study, we determined whether LPS challenge at different stages of gestation modulates the levels of selected ABC transporters in the placenta, which in turn might alter fetal exposure to potentially harmful substrates. Furthermore, we investigated the maternal plasma and placental levels of lipid fractions and the mRNA expression of key placental lipid transporters in dams exposed to a sublethal LPS dose to elucidate the possible effects of LPS on altering lipid homeostasis related or unrelated to placental ABC transporter-mediated lipid exchange. We hypothesized that LPS exposure modifies the expression of key placental ABC and lipid transporters, as well as maternal and placental lipid homeostasis in a gestational age-dependent manner.

## Materials and Methods

### Animal Experiments and Study Design

This study was approved by the Animal Care Committee of the Health Sciences Center, Federal University of Rio de Janeiro (CEUA-190/13) and registered within the Brazilian National Council for Animal Experimentation Control. The study complied with the “Principles of Laboratory Animal Care” generated by the National Society for Medical Research and the United States. National Academy of Sciences Guide for the Care and Use of Laboratory Animals.

Virgin female and male C57BL/6 mice (8–10 weeks of age) were housed in a temperature-controlled room (23°C) on a 12/12h light/dark cycle, with free access to fresh food and water. Female mice in the oestrous phase (identified by vaginal cytology) were time-mated with C57BL/6 males and assigned (gestational day (GD) 0.5) to different groups. Lipopolysaccharide (Sigma, *E. coli* 055:B5; 150 μg/kg; intraperitoneal injection, i.p., in a single dose) or vehicle (i.p. injection of a single dose) was administered to mice in mid- (GD14.5/15.5) or late- (GD17.5/18.5) pregnancy for 24 or 4 h, respectively. Animals were euthanized at GD15.5 or GD18.5 at the end of the 4 or 24 h treatments with a single dose of LPS/vehicle, and maternal blood, fetuses and placentae were collected. Supplementary Figure 1 summarizes the design of the study. Of importance, LPS was specifically chosen to mimic bacterial infection during pregnancy, because LPS induction of Tlr-4 activation by intrauterine microbes poses the greatest risk for pregnancy complications such as HCA and PTB (Conti et al., 2015; Firmal et al., 2020).

Fetal and placental tissues were weighed, and three placentae per litter were selected for further study. The selection was based on placentae with weights closest to the mean, an approach that we and others have used previously (Festing, 2006; Coan et al., 2008; Bloise et al., 2012; Fontes et al., 2019; Connor et al., 2020). The LPS dose (150 μg/kg) was selected because it has previously been shown to cause an acute maternal inflammatory response with less than 50% fetal death in mid-pregnancy at 4 h after treatment (Bloise et al., 2013).

### Quantitative PCR

Total placental RNA was extracted using TRIzol reagent according to the manufacturer’s instructions (Life Technologies, CA, United States). The total RNA concentration was assessed using a nanophotometer (Implen, Munchen, Germany), and samples with RNA purity (260/280 absorbance) ratios ranging between 1.8 and 2.0 and with proven RNA integrity (confirmed through gel electrophoresis) were included in the study. Total RNA (1 μg) was reverse transcribed into cDNAs using the High Capacity cDNA Reverse Transcription Kit (Applied Biosystems, São Paulo, Brazil) according to the manufacturer’s instructions.

The mRNA levels of selected ABC transporters, lipid metabolism-related genes, proinflammatory cytokines and chemokines (Supplementary Table 1) were evaluated using qPCR according to the manufacturer’s recommendations (EVAGREEN; Solis Byodine, EUA) and using the Master Cycler Realplex system (Eppendorf, Germany) with the following cycling conditions: combined initial denaturation steps at 50°C (2 min) and 95°C (10 min), followed by 40 cycles of denaturation at 95°C (15 s), annealing at 60°C (30 s) and extension at 72°C (45 s). Relative gene expression was quantified using the 2^−ΔΔCq^ method (Livak and Schmittgen, 2001). Assays with 95–105% efficiency were considered acceptable.

Gene expression was normalized to the geometric mean of selected reference genes in each experimental group, which exhibited stable expression levels following LPS challenge (Supplementary Table 1). The geometric mean expression of *B2m* and *β-actin* genes was used to normalize mRNA expression at GD15.5, whereas the geometric mean expression of *Gapdh* and *Ywhaz* reference genes was used to normalize mRNA expression at GD18.5. Intron-spanning primers, reverse transcriptase-negative samples and a melting curve analysis obtained for each qPCR were used to exclude DNA contamination.

### Histological and Immunohistochemical Staining

Placental discs were fixed with 4% buffered paraformaldehyde, dehydrated with increasing concentrations of ethanol, diaphanized in xylene, embedded in paraffin and sectioned (5 μm) using a Rotatory Microtome CUT 5062 (Slee Medical GmbH, Germany) for periodic acid-Schiff (PAS) staining and immunohistochemistry as previously described (Fontes et al., 2019).

Briefly, PAS staining was performed by oxidizing placental sections with 0.5% periodic acid (Sigma-Aldrich, Missouri, United States) for 15 min. Sections were washed with distilled water and incubated (10 min at room temperature) with Schiff’s reagent (Merck, Germany), followed by hematoxylin (Proquímios, Rio de Janeiro, Brazil) staining. The junctional zone (Jz) interface between the maternal and fetal placental cellular components was visually identified, and the area of each region of interest, the Jz and the labyrinth zone (Lz), was measured using ImageJ software (National Institutes of Health, Maryland, United States).

Immunohistochemistry was performed by incubating sections with Tris-EDTA buffer (pH 9.0) for 15 min, followed by immersion in sodium citrate buffer (pH 6.0) for 8 min (in a microwave). Sections were immersed in a bovine serum albumin (BSA3% - in PBS) solution to block nonspecific antibody binding sites and then incubated with primary antibodies against P-glycoprotein (P-gp-1:500; Santa Cruz Biotechnology, Texas, United States), breast cancer resistance protein (Bcrp-1:100; Merck Millipore, Massachusetts, United States) or ABC sub-family G member 1 (Abcg1, 1:200; Abcam Plc, United Kingdom) overnight at 4°C. BSA (3% - in PBS) solution was incubated with negative control sections instead of primary antibodies. Sections were then washed with PBS (3 × 5 min) and incubated with a biotin-conjugated secondary antibody (SPD-060 - Spring Bioscience, California, United States) for 1 h followed by an incubation for 1 h with streptavidin (SPD-060 - Spring Bioscience, California, United States). The reaction was halted with 3, 3-diaminobenzidine (SPD-060 - Spring Bioscience, California, United States) followed by hematoxylin (Proquímios, Brazil) staining.

Digital images of histological staining were acquired using a high-resolution Olympus DP72 camera (Olympus Corporation, Japan) attached to the Olympus BX53 microscope (Olympus Corporation, Japan). P-gp, Bcrp and Abcg1 staining were quantified using Image-Pro Plus 5.0 software (Media Cybernetics, Maryland, United States), where the percentage of stained tissue area was calculated and negative spaces were excluded. In each experimental group, 30 digital images per placenta (15 digital images for each labyrinthine and spongiotrophoblast area) were evaluated (Fontes et al., 2019).

### Lipid Analysis

Lipid extraction and analysis were performed using the method described by Bligh and Dyer (1959) with some modifications. Maternal plasma (30 μl) and placentae (10 mg) were separately mixed with a solution containing chloroform (1 ml), methanol (2 ml) and water (0.8 ml) with intermittent shaking. After 2 h, the solution was centrifuged (1,500 g, 20 min, 4°C; Sorvall RC-5b; Sorvall Centrifuge, Newtown, CT, United States), the supernatant was collected, and a water-chloroform solution (1:1 v/v) was added. The mixture was shaken and centrifuged (1,500 g, 20 min, 4°C), and the organic phase was removed and dried under nitrogen gas. The lipid classes were separated by one-dimensional thin layer chromatography (TLC) for neutral lipids using a solution containing hexane, diethyl ether and acetic acid (60:40:1 v/v). Plates were immersed in a solution composed of 3% CuSO4 and 8% H3PO4 (v/v; 10 s) and then heated (110°C, 10 min; Ruiz and Ochoa, 1997). Thin layer chromatography plates were analyzed by densitometry using ImageMaster Total Lab 4.1 software (Total Lab Ltd., Newcastle, United Kingdom). Standards for each lipid species were used to identify different lipid classes (Sigma-Aldrich, Sao Paulo, Brazil). Their IDs and catalogue numbers are as follows: triacylglycerol, 1, 3-dipalmitoyl-2-oleoylglycerol, #D2157; cholesteryl ester, cholesteryl oleate, #C9253; free fatty acids, palmitic acid, #76119; cholesterol, #C8667; and monoacylglycerol, #M2140.

A TLC plate with a standard curve for each of the lipid species was developed to quantify the lipid classes. Densitometric units from the standard curve were compared with densitometric units from the samples (which were already normalized to the unit of tissue or amount of plasma). Thin layer chromatography plates were developed in the mobile phase for 1 h. Supplementary Figures 2, 3 show the TLC plates with plasma and placenta samples from different groups.

### Measurement of Plasma Cytokine and Chemokine Levels

Maternal plasma was collected by cardiac puncture, transferred into heparinized tubes on ice, centrifuged (1,077 g, 15 min) and frozen (−80°C). The levels of interleukin (Il)-1β, Il-6, monocyte chemoattractant protein-1 (Mcp-1/Ccl2) and chemokine (C-X-C motif) ligand 1 (Cxcl1) were assessed using the MILLIPLEX-MAP kit Cytokine/Chemokine Magnetic Bead Panel (MCYTOMAG-70K, Merck Millipore, Germany) according to the manufacturer’s recommendations. Fluorescence intensity was detected using a Luminex 200^™^ system (Merck Millipore, Massachusetts, United States).

### Statistical Analysis

Normality tests were applied followed by Student’s *t*-test or the nonparametric Mann–Whitney test to compare two variables. Pregnancy parameters were evaluated using the mean value of placentae/fetuses in each litter and not individuals (Festing, 2006). For qPCR, PAS/immunohistochemistry, lipid fraction analysis and cytokine/chemokine measurements, three placentae with the closest weight to the mean placental weight within each litter were selected, i.e., one placenta w. Thus, “n” represents the number of litters (Festing, 2006; Coan et al., 2008; Bloise et al., 2013; Fontes et al., 2019; Connor et al., 2020). Values for all data are presented as the means ± SEM. GraphPad Prism 6 software (GraphPad Software, Inc., San Diego, CA, United States) was used to conduct statistical analyses, and differences were considered significant when *p* < 0.05.

## Results

### Acute Sublethal Effects of LPS on Pregnancy Outcomes

The sublethal LPS treatment elicited different pregnancy outcomes that varied according to the time of exposure and gestational age. At GD15.5, LPS at 4 h induced the death of 26% of fetuses, whereas at 24 h, an 84% fetal death rate was observed. Conversely, the same LPS dosage induced only 1 and 2% fetal death rates at GD18.5 after 4 h and 24 h of LPS exposure, respectively. However, a sublethal LPS treatment for 24 h in late pregnancy (GD18.5) induced a 64% increase in early labour compared to controls, i.e., fourteen of twenty-two dams exhibited signs of labour within 24 h (Table 1), i.e., exhibited the presence of one or more offspring (live or dead) in the cage, or exhibited abrupt weight loss concomitant with signs of maternal cannibalism in the cage (McCarthy et al., 2018) and, therefore, were not included in the study. Importantly, the average gestation length for C57BL/6 mice is 19.25 days (range GD18-22; Murray et al., 2010; Fontes et al., 2019), whereas PTB in C57BL/6 mice may occur prior to GD18 (Fontes et al., 2019). Since we were unable to determine the precise birth time within the 24 h limit of LPS treatment (i.e., GD17.5 or GD18.5) in our cohort, we opted to designate this mode of labour as early rather than preterm as a precaution. Of importance, the high percentage of fetal death at GD15.5 and the induction of early labour at GD18.5 prevented us from conducting further placental analysis in the 24 h groups. Thus, all following analyses were performed in tissues from fetuses that did not show signs of death and from dams that did not undergo early labour.

**Table 1:** Pregnancy outcomes following sublethal LPS challenge in mid and late pregnancy.

LPS injection	Groups	Exposure (h)	N (Dams)	Early labour %	Fetal death %	Fetal weight (mg)	Placental weight (mg)	F:P ratio
E15.5	Control	4	8	0	10(8/81)	410 ± 30	95 ± 2	4.3 ± 0.35
	LPS		8	0	36(19/53)	400 ± 30	90 ± 3	4.3 ± 0.12
E14.5	Control	24	6	0	3(1/30)	–	–	–
	LPS		4	0	87(20/23)	–	–	–
E18.5	Control	4	9	0	0(0/35)	1.149 ± 50	80 ± 0.9	14.16 ± 0.6
	LPS		10	0	1(1/68)	1.145 ± 40	90^*^ ± 2.0	12.80 ± 0.64
E17.5	Control	24	12	0	2.5(2/80)	1.130 ± 50	82 ± 0.9	13.39 ± 0.54
	LPS		22	64(14/22)	2.0(1//51)	950^*^ ± 60	92^*^ ± 3	10.72^**^ ± 0.72

Statistics: Student’s *t*-test.

* *p* < 0.05;

** *p* < 0.01.

### Maternal and Placental Proinflammatory/Morphological Responses to Acute Sublethal LPS Exposure

**Figure 1:**
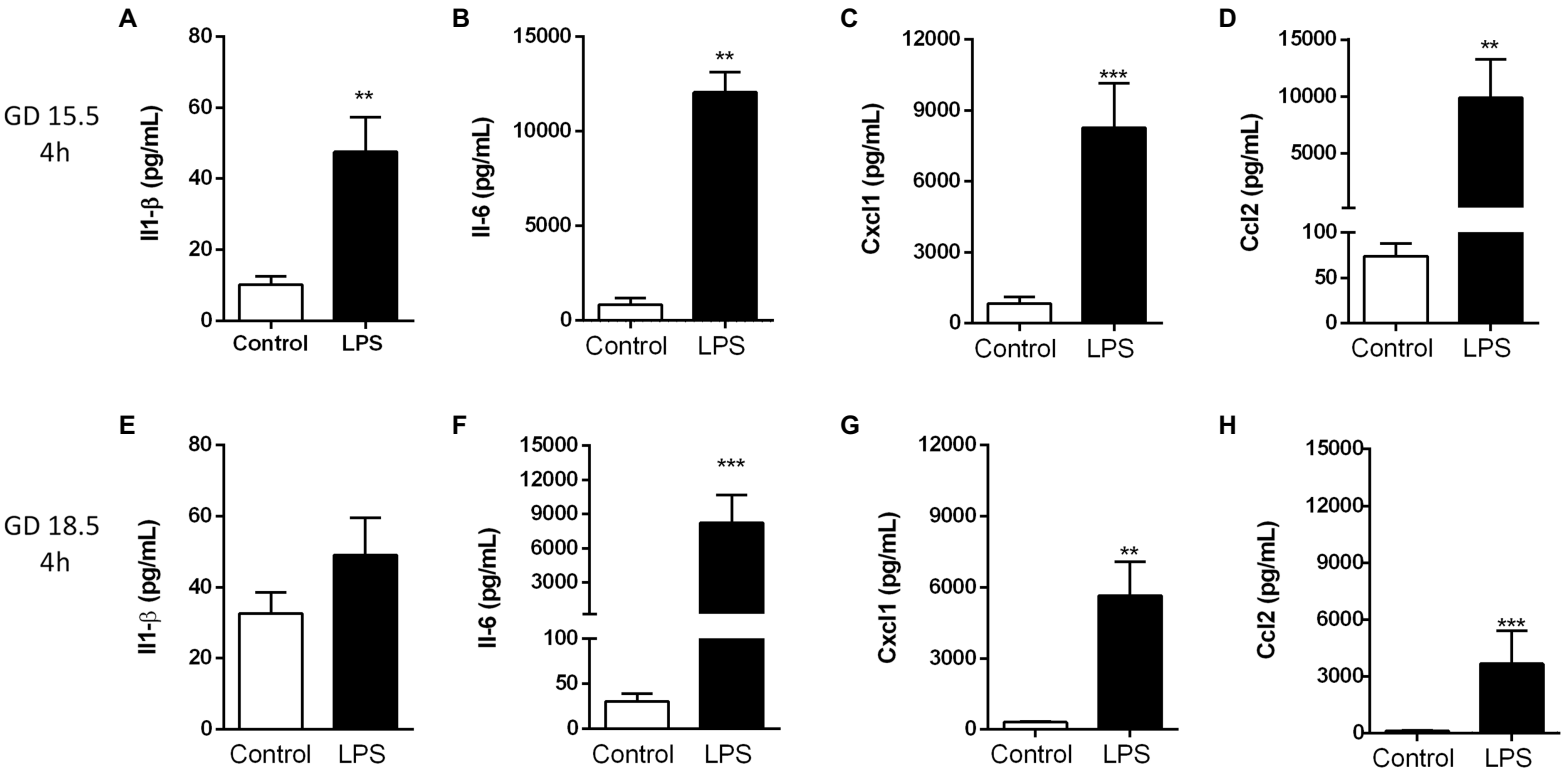
Lipopolysaccharide (4 h) elicited an acute maternal inflammatory response at GD 15.5 and 18.5. Maternal plasma levels of Il-1β **(A,E)**, Il-6 **(B,F)**, Cxcl1 **(C,G)** and Ccl2 **(D,H)** in dams at GD15.5 (control group, *n* = 8, LPS group, *n* = 8) and GD18.5 (control group, *n* = 9, LPS group, *n* = 10), respectively. Statistical analysis: unpaired Student’s *t*-test (GD15.5 and 18.5: Il1-β and Ccl2) or Mann-Whitney nonparametric test (GD15.5: Il-6 and Cxcl1; GD 18.5: Il-6). ^**^p < 0.01 and ^***^p < 0.001.

Maternal plasma levels of cytokines/chemokines associated with the pathogenesis of PTB, including Il-1β, Il-6, chemokine (C-X-C motif) ligand 1 (Cxcl1) and Mcp-1/Ccl2 (Khan and Hay, 2015; Hayward et al., 2016), were assessed in dams following sublethal LPS exposure (4 h) at GD15.5 and GD18.5. At GD15.5, maternal plasma levels of Il-1β (*p* < 0.01), Il-6 (*p* < 0.01), Cxcl1 (*p* < 0.0001) and Ccl2 (*p* < 0.01) were increased compared to the control group (Figures 1A–D). At GD18.5, we observed increased maternal plasma Il-6 (*p* < 0.001), Cxcl1 (*p* < 0.01) and Ccl2 levels (*p* < 0.001; Figures 1F–H), (LPS) challenge whereas Il-1β levels remained unchanged (Figure 1E).

**Figure 2:**
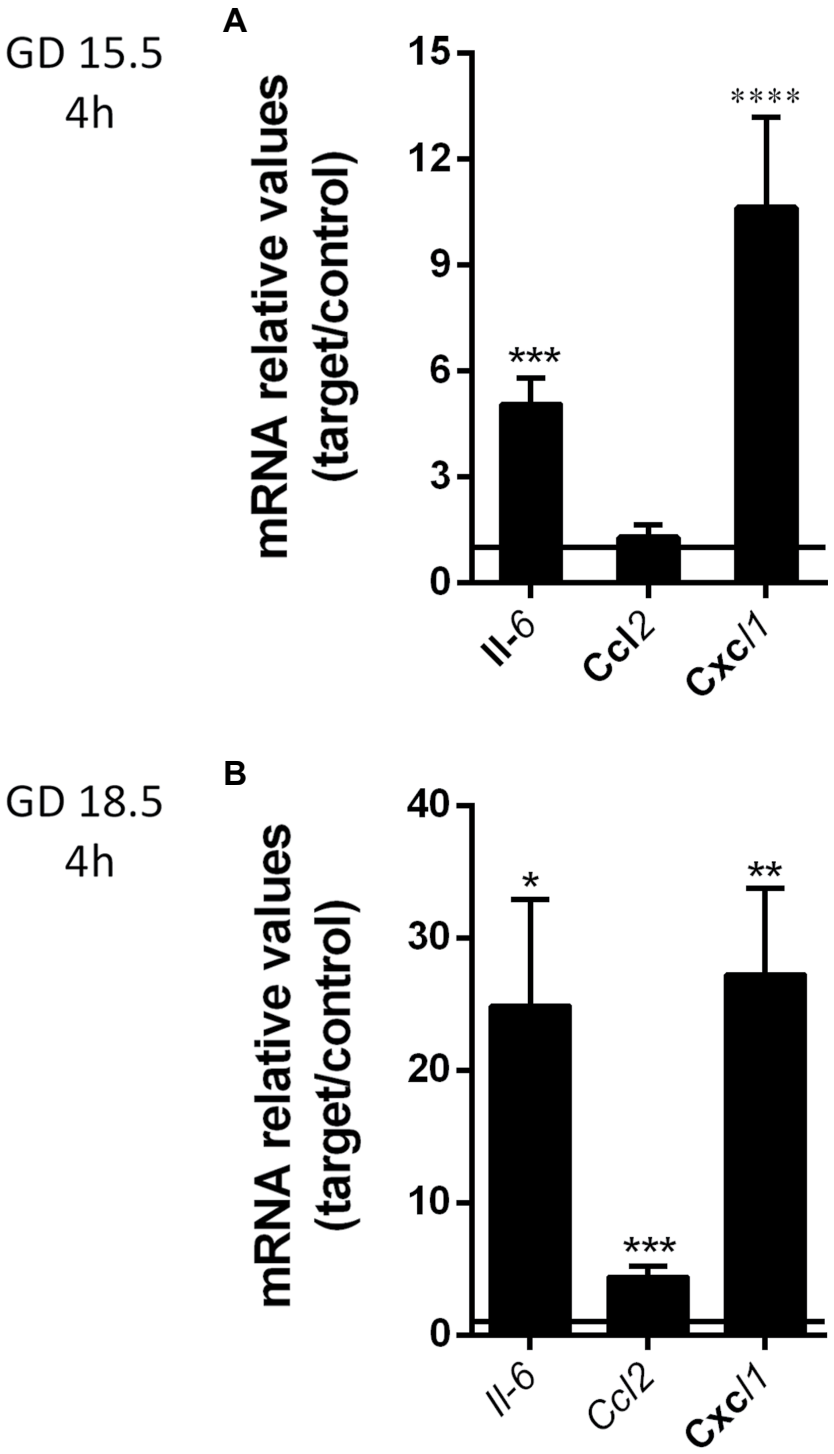
Lipopolysaccharide insult (4 h) elicited an increase in selected placental cytokine/chemokine levels at GD 15.5 and 18.5. mRNA levels of placental *Il6*, *Ccl2* and *Cxcl1* at GD 15.5 **(A)** control group, *n* = 8, LPS group, *n* = 8 and GD 18.5 **(B)** control group, *n* = 9, LPS group, *n* = 10, 4 h after LPS exposure. Gene expression was normalized to the levels of the reference genes *B2m* and *βactin* **(A)**, or *Gapdh* and *Ywhaz* **(B)**. Statistical analysis: GD 15.5: Student’s *t*-test and GD 18.5: Student’s *t*-test. ^*^p < 0,05; ^**^p < 0.01; ^***^p < 0.001; and ^****^p < 0.0001. Line shows the expression levels of the control group.

**Figure 3:**
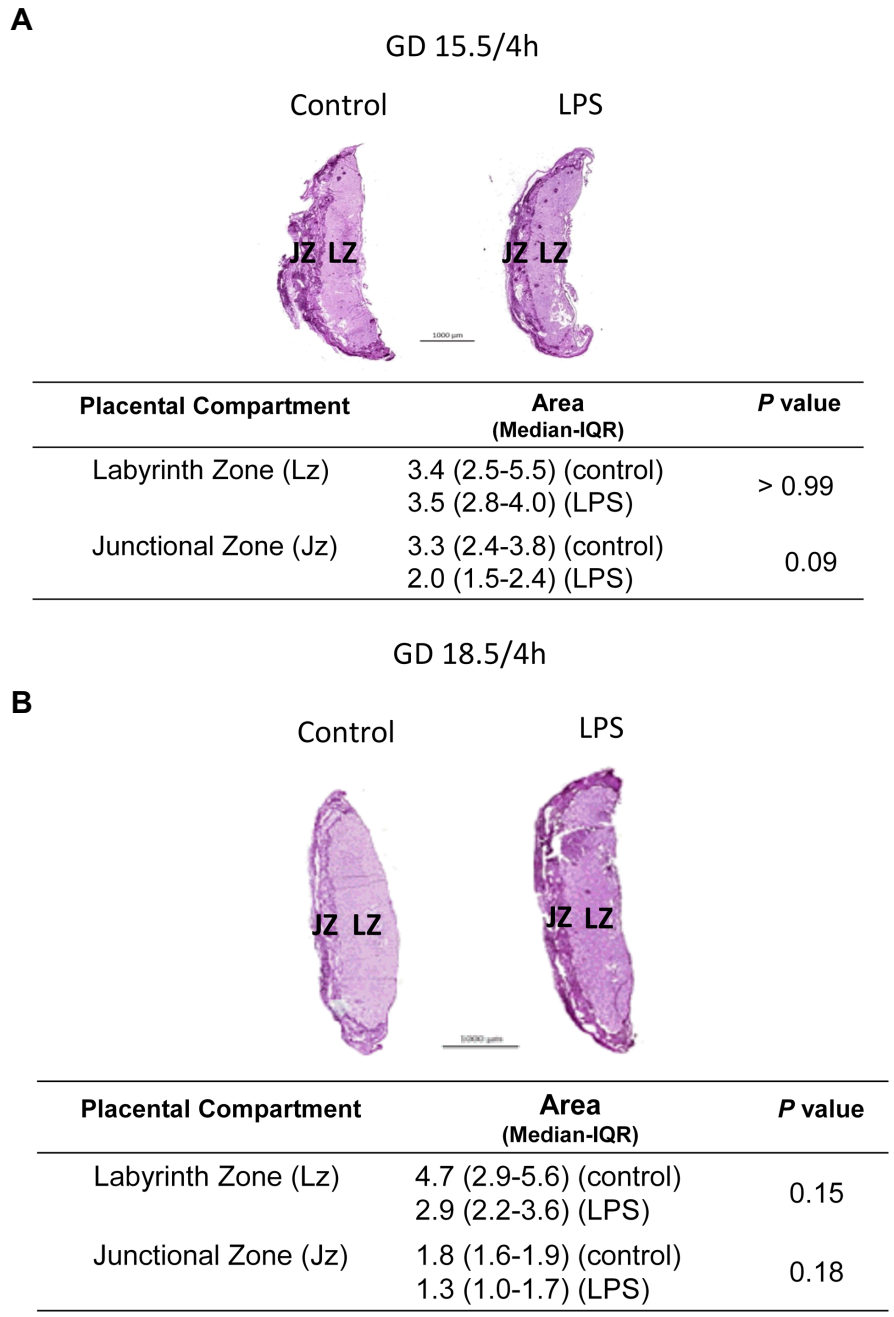
Lipopolysaccharide (4 h) does not alter placental gross morphology. Analysis in the placental proportions of labyrinth (Lz) and junctional zone (Jz) of dams at GD15.5 **(A)** and 18.5 **(B)**, 4 h after LPS exposure. Periodic acid-Schiff staining of control and LPS-treated mice evaluating Lz and Jz areas. *n* = 5/group. Statistical analysis: Mann-Whitney nonparametric test.

In the placenta, the expression of the Il-6 and Cxcl1 (*p* < 0.05) mRNA was significantly increased following LPS challenge at GD 15.5 and GD 18.5 (Figure 2). In contrast, Ccl2 mRNA expression was unchanged at GD15.5, but increased at GD18.5 (*p* < 0.0001; Figure 2B). Placental weight was decreased 4 h after LPS exposure at GD18.5, compared to controls (Table 1). This led us to investigate whether LPS would elicit changes in the placental proportions of Lz and Jz. No differences in gross placental morphology, including changes in Lz and Jz areas, were identified in any of the groups investigated (Figure 3).

### Gestational Age-Dependent Sublethal LPS Effects on Placental ABC Transporters

**Figure 4:**
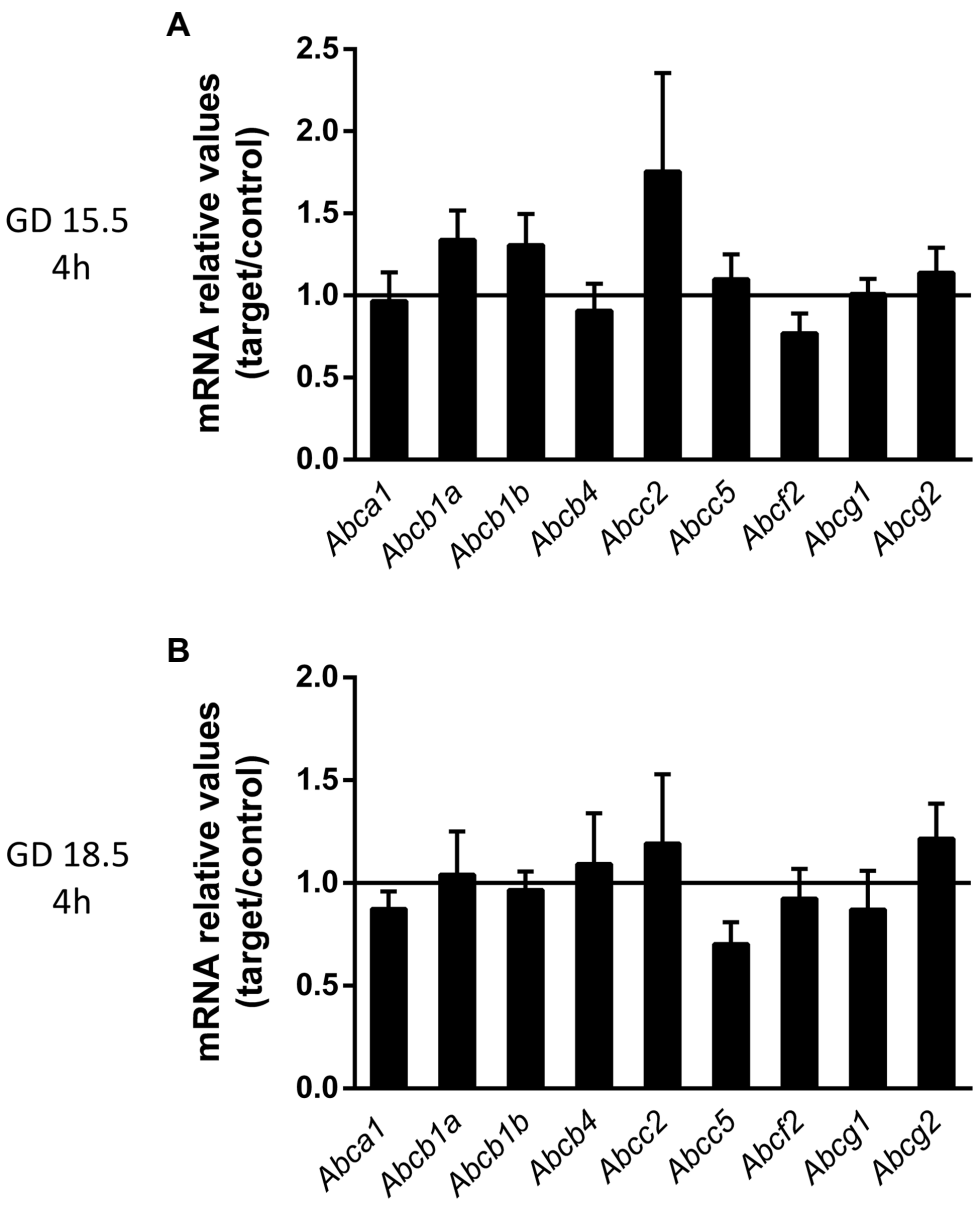
Sublethal LPS challenge (4 h) does not alter the expression of key ABC transporter genes at GDs 15.5 and 18.5. **(A)** Placental levels of the *Abca1*, *Abcb1a*, *Abcb1b*, *Abcb4*, *Abcc2*, *Abcc5*, *Abcf2*, *Abcg1*, and *Abcg2* mRNA at GD15.5 **(A)** and at GD18.5 **(B)**, 4 h after sublethal LPS administration. 15.5/4 h: *n* = 8 (control group); *n* = 8 (LPS group). 18.5/4 h: *n* = 9 (control group); *n* = 10 (LPS group). Gene expression was normalized to the levels of the reference genes **(A)** *B2m* and *βactin* or **(B)** *Gapdh* and *Ywhaz*. Statistical analysis: GD 15.5: Student’s *t* test and GD 18.5: Student’s *t*-test (*Abcb1b*, *Abcb4*, *Abcc5*, *Abcf2*, and *Abcg1*) and Mann-Whitney nonparametric test (*Abca1*, *Abcb1a*, *Abcc2*, and *Abcg2*). Line shows the expression levels of the control group.

To investigate how bacterial infection impacts placental efflux transport potential, we investigated the expression of the of *Abca1*, *Abcb1a*, *Abcb1b*, *Abcb4*, *Abcc2*, *Abcc5*, *Abcf2*, *Abcg1* and *Abcg2* mRNAs in the mouse placenta at GD15.5 or GD18.5 following LPS challenge (4 h). These specific ABC transporter genes were selected based on evidence showing their sensitivity to infection in other models and/or based on their importance to placental and yolk sac barrier function (Bloise et al., 2016; Do Imperio et al., 2018; Fontes et al., 2019; Martinelli et al., 2020a,b). We did not observe significant differences after LPS treatments (4 h) at GD15.5 and GD18.5 (Figure 4).

**Figure 5:**
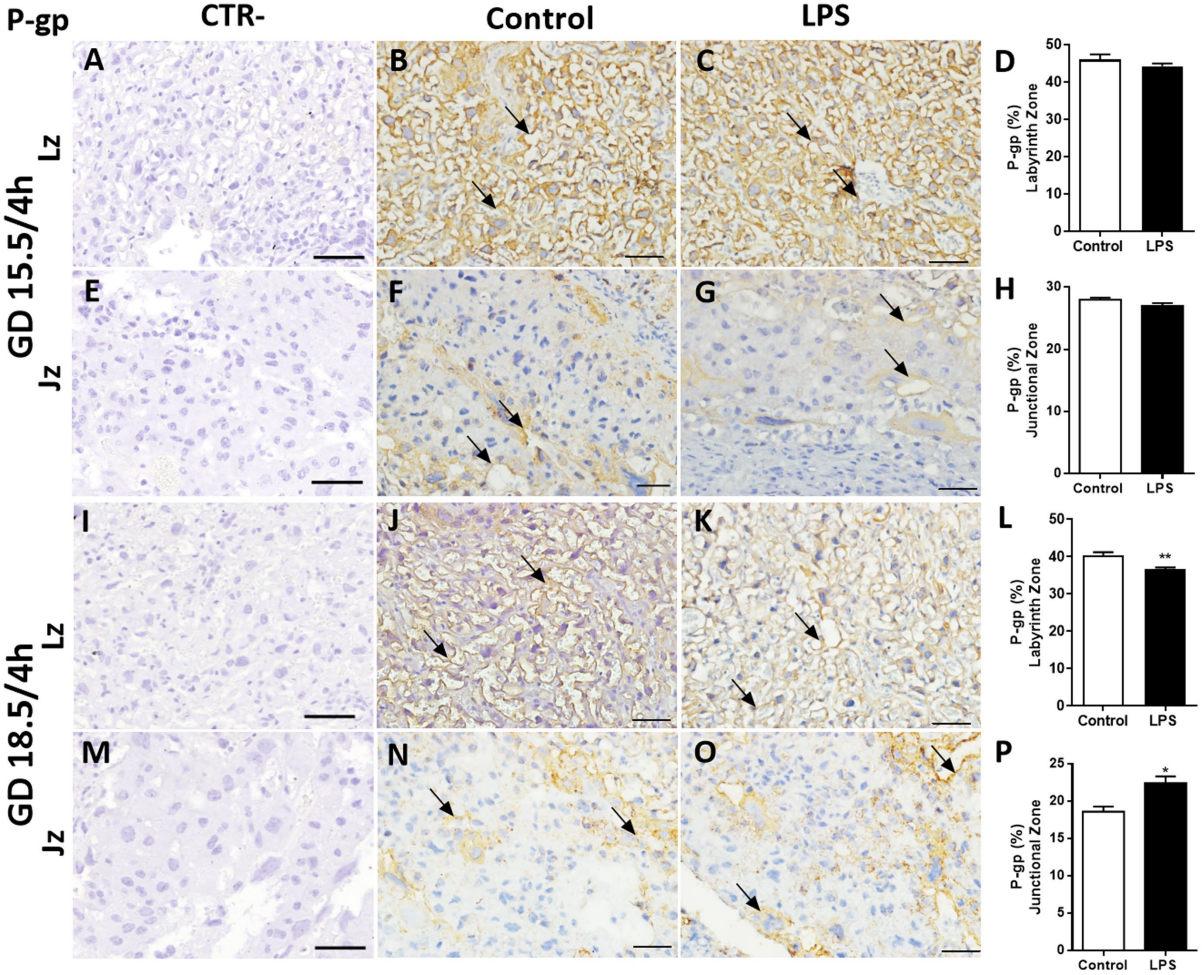
Lipopolysaccharide challenge modulates P-gp protein in the mouse placenta. Representative photomicrographies of immunohistochemistry (arrows) and semiquantitative analysis of P-gp in the labyrinth zone (Lz) and junctional zone (Jz) at GD 15.5/4 h **(A–H)** and GD 18.5/4 h **(I–P)** after LPS administration (4 h). Graphs represent the % of positively stained cells. *n* = 5/group. Scale bar = 50 μm). Statistical analysis: Student’s *t*-test. ^*^p < 0,05; ^**^p < 0.01.

P-gp and Bcrp are key multidrug resistance transporters that have been shown to play roles in fetal protection, whereas the lipid transporter Abcg1 is important for fetal lipid transfer. Therefore, these three ABC transporters were specifically chosen for further examination. P-gp immunostaining was detected in the cellular membrane of labyrinthine cells, with variable staining in the cellular membrane and cytoplasm of spongiotrophoblast cells (Figure 5). Using a semiquantitative analysis, we identified no differences in P-gp staining after LPS administration (4 h) at GD. 15.5 in the Lz and Jz (Figures 5A–H). Reduced P-gp staining intensity was observed in the Lz at GD18.5 (*p* < 0.01) after LPS administration (4 h), compared to controls (Figures 5I–L). In contrast, P-gp intensity in spongiotrophoblast cells was higher at GD18.5 (*p* < 0.05) 4 h after LPS exposure (Figures 5M–P).

**Figure 6:**
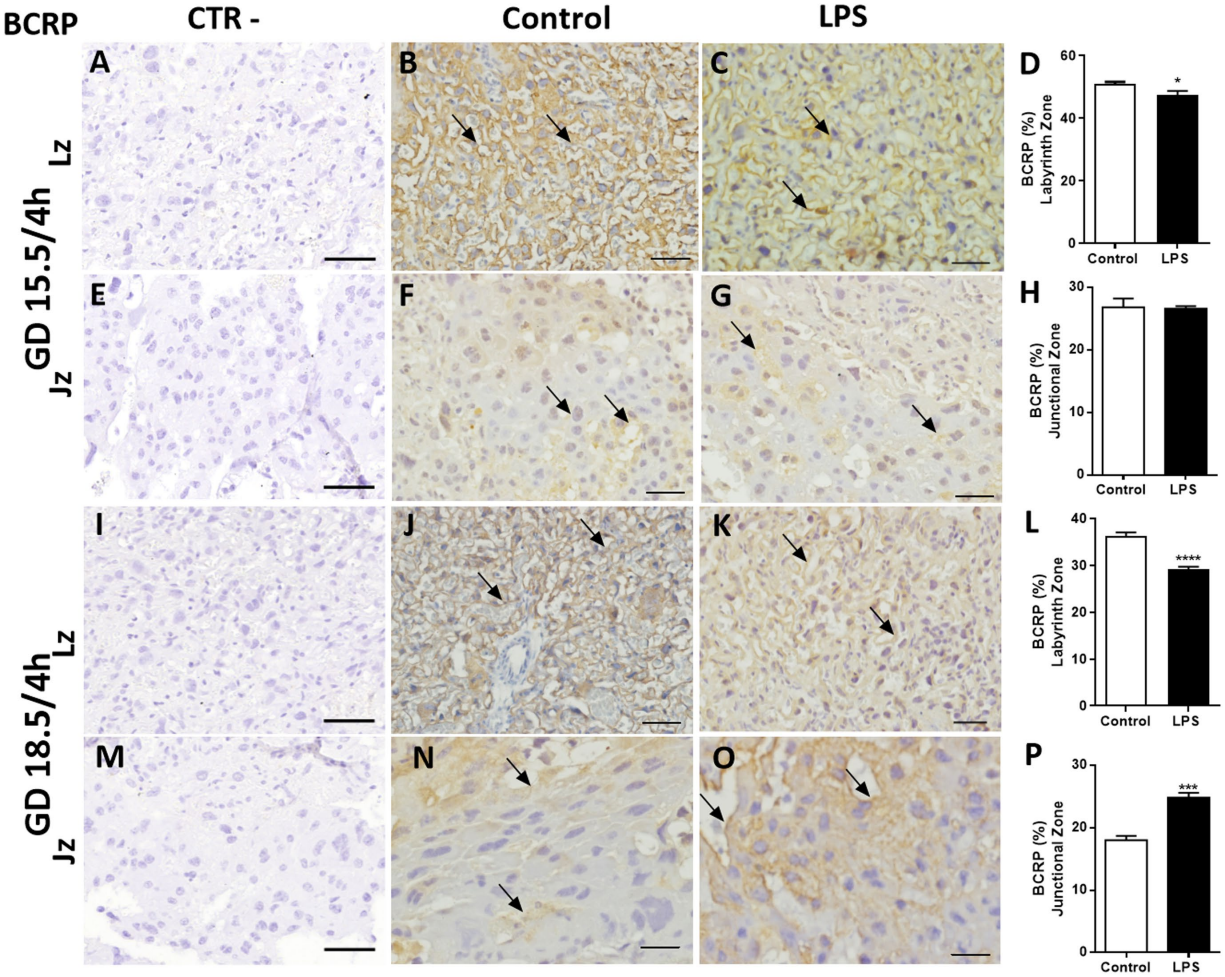
Lipopolysaccharide challenge modulates breast cancer-related protein (Bcrp) protein in the mouse placenta. Representative photomicrographies of immunohistochemistry (arrows) and semiquantitative analysis of Bcrp in the labyrinth zone (Lz) and junctional zone (Jz) at GD 15.5/4 h **(A–H)** and GD 18.5/4 h **(I–P)** after LPS administration (4 h). Graphs represent the % of positively stained cells. *n* = 5/group. Scale bar = 50 μm). Statistical analysis: Student’s *t*-test. ^*^p < 0,05; ^***^p < 0.001; ^****^p < 0.0001.

Breast cancer-related protein exhibited a similar placental distribution pattern, but with faint and variable nuclear staining in labyrinthine cells. Generally, greater Bcrp cytoplasmic labelling was observed in labyrinthine and spongiotrophoblast cells compared to P-gp-labeled placental cells (Figure 6). The Bcrp staining intensity in the Lz was reduced at GD15.5 (*p* < 0.05) and at GD 18.5 (*p* < 0.0001; Figures 6A–D,I–L). We identified no differences in Bcrp staining after LPS administration (4 h) at GD. 15.5 in the Lz and Jz (Figures 6E–H). In contrast, Bcrp intensity in Jz were higher at GD18.5 (*p* < 0.05) 4 h after LPS exposure (Figures 6M–P).

**Figure 7:**
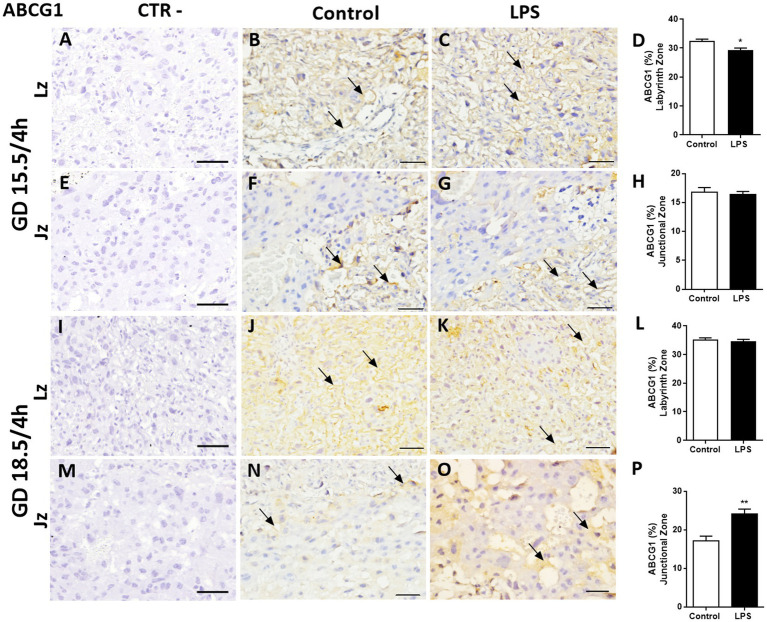
Lipopolysaccharide challenge modulates ABC sub-family G member 1 (Abcg1) protein in the mouse placenta. Representative photomicrographies of immunohistochemistry and semiquantitative analysis of Abcg1 in the labyrinth zone (Lz) and junctional zone (Jz) at GD 15.5/4 h **(A–H)**, GD 18.5/4 h **(I–P)** after LPS administration (4 h). Graphs represent the % of positively stained cells. *n* = 5/group. Scale bar = 50 μm). Statistical analysis: Student’s *t*-test. ^*^p < 0.05; ^**^p < 0.01.

The lipid transporter Abcg1 was predominantly localized to cellular membranes in the Lz and Jz, with some variable cytoplasmic staining throughout these layers (Figure 7). A lower Abcg1 staining intensity was observed in labyrinth cells at GD 15.5 (*p* < 0.05), whereas at GD18.5, it remained unchanged (Figures 7A–D,I–L). No differences in the levels of Abcg1 in spongiotrophoblast cells of the Jz were identified at GD15.5 after LPS treatment (Figures 7E–H). However, when we evaluated at GD 18.5, Abcg1 levels were higher after LPS treatment (*p* < 0.01, Figures 7M–P).

Supplementary Figures 4–6 depict higher magnification photomicrographies of placental P-gp, Bcrp and Abcg1 staining, respectively.

### LPS Alters Maternal and Placental Lipid Homeostasis Throughout Pregnancy

Since we observed an inhibitory effect of LPS on Abcg1 expression (a lipid transporter) in the Lz, we investigated the impact of sublethal LPS exposure (4 h) on the maternal plasma and placental levels of various lipid classes (triacylglycerol, free fatty acids, cholesterol ester, cholesterol, monoacylglycerol, and phospholipids) at GDs 15.5 and 18.5.

We observed significant alterations in placental lipid levels following sublethal LPS challenge. Triacylglycerol, free fatty acid, cholesterol ester and free cholesterol levels were decreased at GD15.5 compared to the control groups (*p* < 0.01, Table 2), whereas triacylglycerol, free fatty acid, free cholesterol, monoacylglycerol and phospholipid levels were increased at GD18.5 compared to the control groups (*p* < 0.05, Table 3). The levels of the lipid classes monoacylglycerol and phospholipid remained unchanged at GD15.5 (Table 2), whereas cholesterol ester levels did not exhibit alterations at GD18.5 (Table 3).

**Table 2:** LPS challenge modulates placental lipid fractions at GD15.5 after LPS administration.

Placental lipid fractions	Groups	Mean ± SEM (μg/mg)	*p*
Triacylglycerol	Control	0.05 ± 0.002	0.0001^***^
	LPS	0.03 ± 0.002	
Free fatty acids	Control	0.014 ± 0.001	0.0043^**^
	LPS	0.008 ± 0.001	
Cholesterol ester	Control	0.18 ± 0.02	0.005^**^
	LPS	0.10 ± 0.01	
Cholesterol	Control	0.05 ± 0.003	0.0043^**^
	LPS	0.03 ± 0.004	
Monoacylglycerol	Control	0.02 ± 0.0002	0.66
	LPS	0.02 ± 0.002	
Phospholipids	Control	0.073 ± 0.003	0.51
	LPS	0.077 ± 0.005	

15.5/4 h: *n* = 6 (control group); *n* = 5 (LPS group). Statistical analysis: Student’s *t*-test.

** *p* < 0.01

*** *p* < 0.001.

**Table 3:** LPS challenge modulates placental lipid fractions at GD18.5 after LPS administration.

Placental lipid fractions	Groups	Mean ± SEM (μg/mg)	*p*
Triacylglycerol	Control	0.04 ± 0.009	0.03^*^
	LPS	0.08 ± 0.008	
Free fatty acids	Control	0.004 ± 0.0005	<0.0001^****^
	LPS	0.03 ± 0.001	
Cholesterol ester	Control	0.23 ± 0.02	0.09
	LPS	0.19 ± 0.01	
Cholesterol	Control	0.026 ± 0.001	<0.0001^****^
	LPS	0.04 ± 0.0008	
Monoacylglycerol	Control	0.03 ± 0.002	0.0002^***^
	LPS	0.04 ± 0.003	
Phospholipid	Control	0.065 ± 0.002	0.04^*^
	LPS	0.071 ± 0.002	

18.5/4 h: *n* = 8 (control group); *n* = 6 (LPS group). Statistical analysis: Student’s *t*-test.

* *p* < 0.05

*** *p* < 0.001

**** *p* < 0.0001.

We detected increased triacylglycerol levels and decreased free fatty acid and cholesterol ester levels in the maternal plasma from the LPS-exposed group at GD15.5 compared to the control group (*p* < 0.001, Table 4). At GD18.5, the triacylglycerol levels were decreased in the LPS group compared to the control group (*p* < 0.05, Table 5). No differences were observed in the levels of free cholesterol, monoacylglycerol and phospholipid classes at either gestational age, whereas the cholesterol ester contents remained unchanged at GD18.5 (Table 5).

**Table 4:** LPS challenge modulates plasma lipid fractions at GD15.5 after LPS administration.

Plasma lipid fractions	Groups	Mean ± SEM (μg/μl)	*p*
Triacylglycerol	Control	0.31 ± 0.02	<0.0001^****^
	LPS	0.56 ± 0.02	
Free fatty acids	Control	0.14 ± 0.01	<0.0001^****^
	LPS	0.06 ± 0.003	
Cholesterol ester	Control	0.31 ± 0.02	0.0003^***^
	LPS	0.19 ± 0.01	
Cholesterol	Control	0.11 ± 0.02	0.59
	LPS	0.13 ± 0.02	
Monoacylglycerol	Control	0.04 ± 0.003	0.095
	LPS	0.06 ± 0.006	
Phospholipid	Control	0.30 ± 0.07	0.26
	LPS	0.38 ± 0.03	

15.5/4 h: *n* = 6 (control group); *n* = 8 (LPS group). Statistical analysis: Student’s *t*-test.

*** *p* < 0.001

**** *p* < 0.0001.

**Table 5:** LPS challenge modulates plasma lipid fractions at GD18.5 after LPS administration.

Plasma lipid fractions	Groups	Mean ± SEM (μg/μl)	*p*
Triacylglycerol	Control	0.76 ± 0.07	0.04^*^
	LPS	0.57 ± 0.03	
Free fatty acids	Control	0.06 ± 0.01	0.44
	LPS	0.08 ± 0.01	
Cholesterol ester	Control	0.16 ± 0.03	0.4
	LPS	0.22 ± 0.07	
Cholesterol	Control	0.12 ± 0.02	0.23
	LPS	0.15 ± 0.02	
Monoacylglycerol	Control	0.07 ± 0.009	0.26
	LPS	0.06 ± 0.007	
Phospholipid	Control	0.19 ± 0.01	0.19
	LPS	0.20 ± 0.008	

18.5/4 h: *n* = 9 (control group); *n* = 7 (LPS group). Statistical analysis: Student’s *t*-test.

* *p* < 0.05.

**Figure 8:**
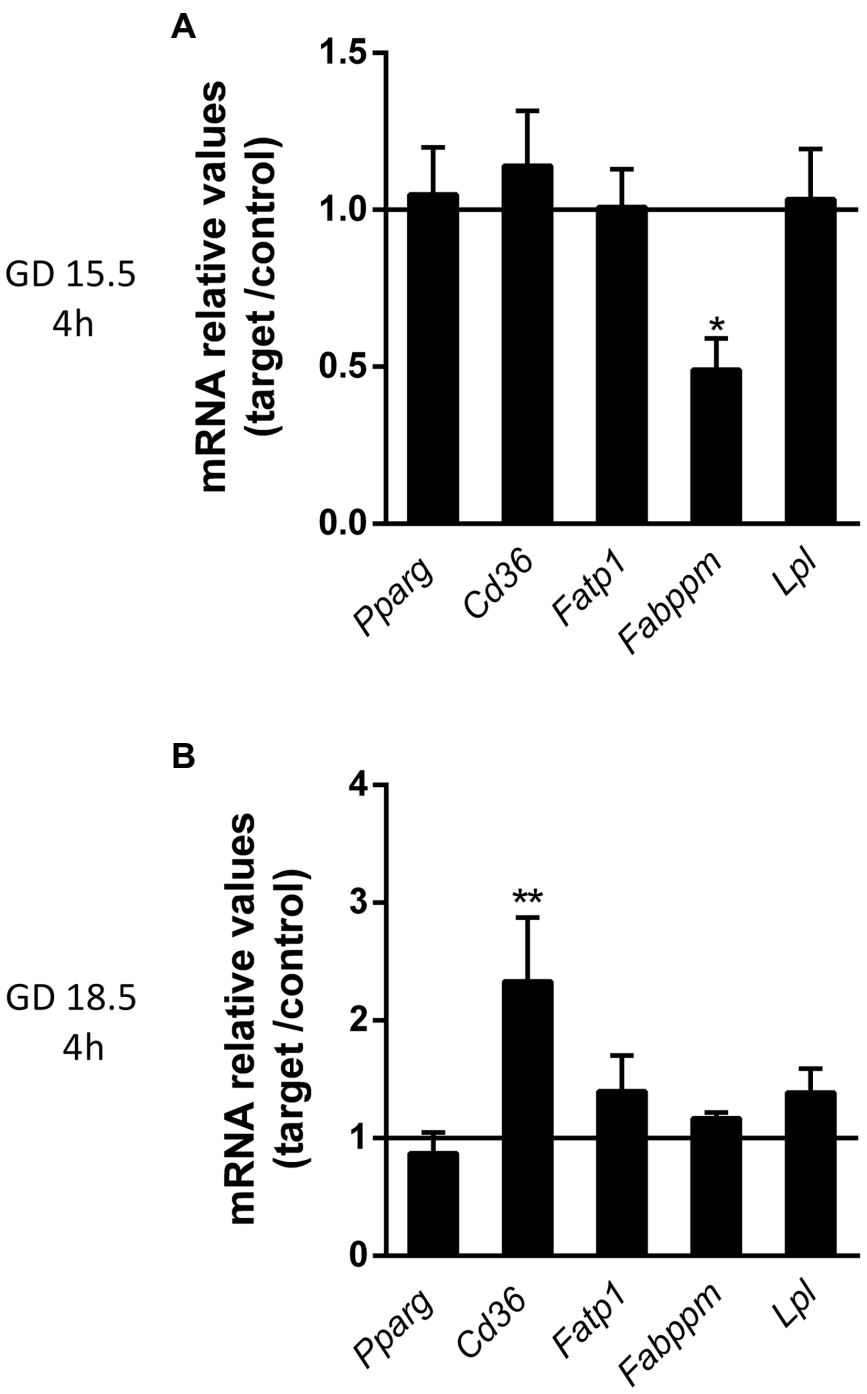
Lipopolysaccharide challenge modulates the mRNA expression of lipid transporters and associated proteins at GDs 15.5 and 18.5, 4 h after the insult. **(A,B)** Quantification of the relative mRNA expression of selected lipid transporters (*Pparg* and *Cd36*) and associated proteins (*Fatp1*, *Fabppm* and *Lpl*) at GD 15.5 and 18.5 4 h after LPS challenge. 15.5/4 h *n* = 8 (control group); *n* = 8 (LPS group). 18.5/4 h: *n* = 9 (control group); *n* = 10 (LPS group). Gene expression was normalized to the levels of the reference genes **(A)** *B2m* and *ß-actin*, or **(B)** *Gapdh* and *Ywhaz*. Statistical analysis: GD 15.5: Student’s *t*-test and GD 18.5: Student’s *t*-test (*Lpl*, *Fabppm*, *Fatp1*, *Pparg*) and Mann-Whitney nonparametric test (*Cd36*). ^*^p < 0.05; ^**^p < 0.01. Line shows the expression levels of the control group.

Placental mRNA expression of key lipid transporters and lipid metabolism-related genes following sublethal LPS treatments was assessed. At GD15.5 (4 h), LPS exposure resulted in lower fatty acid binding protein associated with plasma membrane (*Fabppm*) mRNA levels (*p* < 0.05), whereas at GD18.5 (4 h), increased placental fatty acid translocase (*Fat/Cd36*) levels (*p* < 0.01) were detected (Figures 8A,B). The mRNA expression of other lipid transporters and metabolism-related genes, such as *Pparg*, fatty acid transporter family protein (*Fatp1*) and lipase lipoprotein (*Lpl*), remained unchanged throughout pregnancy.

## Discussion

We have documented the effects of sub-lethal LPS on placental ABC transporters and lipid homeostasis. Lipopolysaccharide caused substantial fetal death at GD15.5 and triggered early labour in late pregnancy (GD18.5) without inducing fetal demise. These effects were likely mediated by distinct maternal and placental cytokine/chemokine responses to LPS throughout pregnancy and were associated with distinct expression profiles of placental efflux and lipid transporters, as well as changes in maternal and placental lipid levels.

Our present findings showing a greater fetal demise susceptibility to LPS at GD15.5 are consistent with previous studies from our group (Bloise et al., 2013). However, here, we also show that sublethal LPS challenge in late pregnancy increased early labour, probably by intensifying the maternal and placental output of labour-inducing cytokines and chemokines. Our data are consistent with one study conducted in rats showing that LPS treatment (24 h) at GD18.5 induced early labour in 55% of pregnancies (Toyama et al., 2015), a percentage similar to the value reported in our study (64%). Furthermore, LPS administration in late gestation decreased the fetal weight, stimulated placental growth and reduced the F:P weight ratio. These changes might indicate an impairment of placental nutrient transport efficiency that prevents fetuses from attaining optimal growth potential (Bloise et al., 2014; Khan and Hay, 2015; Toyama et al., 2015; Hayward et al., 2016) and predisposing them to poor long-term health outcomes (Bloise et al., 2014).

Acute sublethal LPS exposure during pregnancy elicited an intense maternal and placental proinflammatory response, which varied across pregnancy. We observed a marked increase in a number of key proinflammatory factors (IL-6 and Cxcl1 and Ccl2) related to PTB. In contrast, plasma Il-1β levels and placental levels of the Ccl2 mRNA were not consistently affected by LPS, since they did exhibit a gestational age-dependent expression profile. IL-1β, IL-6 and IL-8 are commonly upregulated by infection and induce prostaglandin (PG)E2 and PGF_2α_ production to stimulate myometrial contractility and PTB (Kim et al., 2004; Vrachnis et al., 2012; Khan and Hay, 2015). Ccl2 is subsequently upregulated in the human myometrium during preterm and term labour and recruits infiltrating leukocytes into the myometrium to amplify local inflammation and trigger the onset of labour (Shynlova et al., 2009). Ccl2 also induces the expression of proinflammatory cytokines, prostaglandins and leukotrienes in the myometrium during late pregnancy (Gibb, 1998; Whittle et al., 2000). Importantly, Tlr4 activation induces the myometrial production of Il-1β, Il-6 and Ccl2 *via* nuclear factor-κB (NF-κB) and p38 mitogen-activated protein kinase activation (Chen et al., 2020).

In the present study, LPS stimulated placental Ccl2 expression in a gestational time-dependent manner, suggesting that this cytokine may participate in the pathogenesis of infective chorioamnionitis and the induction of early labour. Furthermore, our data extracted from pregnancies that did not undergo early labour and at an earlier time point (4 h) indicate that a lack of Il-1β upregulation in these pregnancies may have been important in preventing an earlier onset of labour. Future studies comparing the maternal and placental inflammatory responses of pregnancies that did and did not undergo LPS-induced early labour are required to test this hypothesis.

We evaluated the mRNA and protein expression/localization of selected ABC transporters involved in the biodisposition of clinically relevant substrates across pregnancy to understand how a sublethal bacterial infection alters placental efflux transport potential. As shown in our previous study, a sublethal LPS challenge (150 μg/kg) impairs placental P-gp activity at GD15.5 (4 h) in C57BL/6 mice, with no changes in placental *Abcb1a* and *Abcb1b* mRNA levels (Bloise et al., 2013). In the present study, no changes in P-gp-encoding genes (*Abcb1a* and *Abcb1b*) were observed at GD15.5 and GD18.5 (4 h), however, lower labyrinthine P-gp staining intensity at GD18.5 (4 h) was detected. Placental levels of P-gp and its encoding genes are developmentally regulated in both rodents and humans, i.e., expression decreases towards term (Kalabis et al., 2005; Lye et al., 2013) resulting in reduced fetal protection against P-gp substrates in late pregnancy. Based on our results, a late-term bacterial infection may further decrease this already limited P-gp-mediated barrier function. These changes will likely increase the levels of cytokines and chemokines within gestational tissues, causing fetal demise and morbidity and/or inducing preterm/early labour pathways.

The results from the present and previous studies suggest that bacterial infection has the potential to increase fetal accumulation of P-gp substrates (cytokines, chemokines, endogenous and synthetic glucocorticoids, antibiotics, antiretrovirals, etc.; Bloise et al., 2016) both in earlier stages of pregnancy due to impaired placental P-gp activity (Bloise et al., 2013) and in later stages of pregnancy by decreasing labyrinthine-P-gp expression. These changes will likely increase the levels of cytokines and chemokines within gestational tissues, causing fetal demise and morbidity and/or inducing preterm/early labour pathways.

Consistent with our data, P-gp is downregulated in (1) human first trimester placental explants exposed to LPS (Lye et al., 2015), (2) human PTB placentae with chorioamnionitis (Do Imperio et al., 2018), (3) placentae from pregnant malaria-infected C57BL/6 mice exhibiting high rates of PTB (Fontes et al., 2019), and (4) placentae from pregnant ZIKV-infected mice (Andrade et al., 2021). Furthermore, P-gp activity is impaired in the developing fetal blood brain barrier of pregnant C57BL/6 mouse exposed to PolyI:C, a TLR3 viral mimic (Bloise et al., 2017). Together, these studies suggest that lower placental *Abcb1b* and P-gp levels are associated with increased risk of infection-driven PTB (Do Imperio et al., 2018; Fontes et al., 2019) and/or earlier labour and delivery. The effects of lower placental P-gp levels on fetal health and postnatal development require further investigation.

A lower level of Bcrp immunostaining was observed in the Lz at GDs 15.5 and 18.5, following LPS exposure. Placental Bcrp levels have been shown to be downregulated in human first trimester placental explants treated with LPS (Lye et al., 2015), in malaria-infected murine placentae (Fontes et al., 2019) and in HTR8/SVneo (human extravillous trophoblast-like) cells exposed to either LPS or to the TLR8 viral mimic single stranded RNA (ssRNA; Lye et al., 2019). Thus, different infective agents/challenges during pregnancy, including bacterial infection, have the potential to increase fetal accumulation of Bcrp substrates during pregnancy. However, Bcrp was upregulated in human PTB placentae with chorioamnionitis (Do Imperio et al., 2018) which is commonly induced by polymicrobial infection (Conti et al., 2015), and after treatment of trophoblastic cells with PGE2 (Mason et al., 2014), indicating that the nature of infective/inflammatory stimuli determines the trophoblastic-Bcrp modulatory response. Furthermore, in the present study, we focused in the effects of LPS rather than infection of different strains of gram–bacteria related to PTB and chorioamnionitis. It is possible that infection by different bacterial strains or by polymicrobial infection would impose distinct responses in placental cytokine and transporter levels.

Interestingly, the P-gp, Bcrp and Abcg1 staining intensity in the spongiotrophoblast was higher at GD18.5 following LPS (4 h) administration but not different at GD15.5. The role of ABC transporters in spongiotrophoblast cells is far less understood. Breast cancer-related protein immunolocalization has been previously reported in spongiotrophoblasts, which remained unaltered in pregnancies in which the mother underwent nutritional manipulations (Connor et al., 2020). Spongiotrophoblasts comprise the mouse placental Jz and provide structural support for the growth of the labyrinthine villi and limit fetal endothelium overgrowth (Silva and Serakides, 2016). However, very little is known about the possible functions of ABC transporters in the mouse Jz and how infection and inflammation impact Jz function.

Labyrinthine Abcg1 expression was also downregulated by LPS at GD15.5. ABC sub-family G member 1 is a cholesterol and phospholipid efflux transporter predominantly localized to the basolateral membrane of the syncytiotrophoblast and in the endothelium of the fetal capillaries of the human placenta, suggesting that it mediates lipid efflux from the maternal compartment to the fetal compartment (Bloise et al., 2016). However, the directionality of Abcg1-mediated lipid exchange in the mouse placenta has yet to be described, preventing us from postulating on the effects of sublethal LPS exposure on placental Abcg1-mediated biodisposition of cholesterol and other lipids.

We did not detect changes in ABC transporter mRNA expression levels at GD 15.5 and 18.5. It is possible that evaluation of placental mRNA extracted from live fetuses at time points different than 4 h, could potentially detect a transcriptional regulation of these transporters. Furthermore, we observed a general disconnection between placental mRNA and protein levels, which has been frequently reported for ABC transporters in the placenta before (Lye et al., 2013; Petrovic et al., 2015; Do Imperio et al., 2018; Connor et al., 2020). This disconnection may indicate post-transcriptional influences of Tlr-4 activation. In fact, there is evidence of miRNAs regulating P-gp in human placenta with chorioamnionitis (Do Imperio et al., 2018). Alternatevly this disconnection may be derived from the fact that we investigated mRNA levels from whole placental tissue, wheres quantification of protein levels was undertaken in the Lz and Jz compartments.

One limitation of the study is that we have not undertaken functional analysis of placental P-gp, Bcrp and Abcg1 activity following sub-lethal LPS exposure. However, as previously discussed, prior work from our group has demonstrated that LPS treatment (150ug/kg for 4), impaired placental P-gp activity at GD15.5 but not later in pregnancy in C57BL/6 mice (Bloise et al., 2013); and that it occurred without changes in placental *Abcb1a* and *Abcb1b* expression. Of importance, LPS challenge in human cerebral microvascular endothelial cells (hCMEC/D3) altered the expression and activity of P-gp and Bcrp in a dose- and time- dependent manner (Eustaquio Do Imperio et al., 2021). In this context, no studies have investigated whether sub-lethal LPS challenge impact placental expression and function of P-gp, Bcrp and Abcg1, simultaneously. This clearly requires further investigation.

Maternal plasma and placental lipid levels were subsequently investigated to better understand how LPS alters lipid homeostasis at the maternal-fetal interface. Maternal levels of triacylglycerol and free fatty acids were altered after LPS exposure at GD15.5. Similar results have been reported in hepatic tissue following LPS exposure (Liu et al., 2015), which occurs in a gestational time-dependent manner. In fact, relevant alterations in plasma lipoproteins occur during injury or infection (Lewis and Desoye, 2017) or in patients with sepsis who occasionally present with hypertriglyceridemia (Lewis and Desoye, 2017). The higher plasma triacylglycerol levels may be directly related to the inflammatory status in pregnant mice and may be associated with the high rates of fetal death at GD15.5. Nevertheless, higher placental triacylglycerol and free fatty acids levels were detected at GD18.5. This later effect may be an attempt to circumvent the lower fetal weight and one possible mechanism responsible for the higher placental weight observed in this group.

Placental fatty acid transport is modulated by different transport systems, including fatty acid transporter family proteins (FATPs), fatty acid binding proteins (FABPs), fatty acid binding protein associated with plasma membrane (*Fabppm*), lipase lipoproteins (LPL) and *Fat/cd36* translocase located on both apical and basolateral membranes of the trophoblast (Cetin et al., 2012; Daniel et al., 2016). We observed decreased placental *Fabppm* at GD15.5 and increased *Fat/cd36* mRNA expression at GD18.5 after LPS exposure (4 h). This finding may at least partially explain the different patterns of free fatty acid accumulation we observed in the present study. Fetuses (and intrauterine tissues) in pregnancies complicated by maternal bacterial infection may be exposed to higher levels of cytokines/chemokines, drugs and environmental toxins present in the maternal circulation. These changes may also be associated with suboptimal placental lipid storage. Combined, these changes may contribute to the inflammatory PTB pathways and lead to the onset of PTB or early labour.

In conclusion, this sublethal LPS model of bacterial infection during pregnancy may induce an increased risk of fetal death or early labour, depending on the gestational age. Pregnancies with increased risk of fetal death and early labour, due to LPS exposure, may exhibit specific maternal and placental inflammatory responses, altered expression of ABC and lipid transporters and altered maternal and placental lipid - homeostasis.

## Data Availability Statement

The original contributions presented in the study are included in the article/Supplementary Material, further inquiries can be directed to the corresponding author.

## Ethics Statement

This study was approved by the Animal Care Committee of the Health Sciences Center, Federal University of Rio de Janeiro (CEUA-190/13) and registered within the Brazilian National Council for Animal Experimentation Control.

## Author Contributions

MR, CA, GI, GK, SM, EB, and TO-C: conceptualization of experiments. MR, KF, VM, NS, CA, HG, FB, and GK: formal analysis and performed experiments. EB, SM, and TO-C: funding acquisition. EB, SM, GA, and TO-C: project supervision. MR, EB, SM, and TO-C: manuscript draft. KF, VM, NS, CA, HG, GI, FB, and GA: review and editing. All authors contributed to the article and approved the submitted version.

## Conflict of Interest

The authors declare that the research was conducted in the absence of any commercial or financial relationships that could be construed as a potential conflict of interest.

## Publisher’s Note

All claims expressed in this article are solely those of the authors and do not necessarily represent those of their affiliated organizations, or those of the publisher, the editors and the reviewers. Any product that may be evaluated in this article, or claim that may be made by its manufacturer, is not guaranteed or endorsed by the publisher.
