# Supplementary material for: Effect of Sublethal Prenatal Endotoxaemia on Murine Placental Transport Systems and Lipid Homeostasis
Source: Front Microbiol. 2021 Jul 30;12:706499. doi: 10.3389/fmicb.2021.706499 (PMC8363225; doi:10.3389/fmicb.2021.706499)
Supplement: Supplementary file 1 [file Data_Sheet_1.PDF]

Supplementary Material

Supplementary table 1: List of Primers Used in the Present Study.

Gene	Primer Sequences	Reference	Genbank no
Abca1	5’GCAGATCAAGCATCCCAACT3’<br>3’CCAGAGAATGTTTCATTGTCCA5’	(Hirai et al., 2007)	NM_013454.3
Abcb1a	5’GGGCATTTACTTCAAACTTGTCA3’<br>3’TTTACAAGCTTCATTTCCTAATTCAA5’	(Hirai et al., 2007)	NM_011076.3
Abcb1b	5’AAGCCAGTATTCTGCCAAGCAT3’<br>3’CTCCAGACTGCTGTTGCTGATG5’	(Hirai et al., 2007)	NM_011075.2
Abcb4	5’GAAGGGATCTACTTCAGACTCGTT3’<br>3’TCAACTTCAAATTCTTCTGACAGG5’	(Hirai et al., 2007)	NM_008830.2
Abcc2	5’TAATGAGGCGCCGTGGGTGAC3’<br>3’GTCCTGCCCACCACACCGAC5’	(Hirai et al., 2007)	NM_013806.2
Abcc5	5’AAATGTATGCCTGGGTCAAAGC3’<br>3’TGGCGATCACTACCACAATAGG5’	*	NM_013790.2
Abcf2	5’TGTCCACATTATCAACCTCTCCC 3’<br>3’TCACGTTTCCCAATAGCCGAG 5’	*	NM_013853.2
Abcg1	5’GCTCCATCGTCTGTACCATCC3’<br>3’ACGCATTGTCCTTGACTTAGG5’	*	
Abcg2	5’GCCTTGGAGTACTTTGCATCA3’<br>3’AAATCCGCAGGGTTGTTGTA5’	(Merrell et al., 2014)	NM_011920.3
Il6	5’GAGGATACCACTCCCAACAGACC 3’<br>3’AAGTGCATCATCGTTGTTCATACA 5’	(Murakami et al., 2013)	NM_031168.2
Cxcl1	5’ACCCGCTCGCTTCTCTGT 3’<br>3’AAGGGAGCTTCAGGGTCAAG 5’	(Murakami et al., 2013)	NM_008176.3
Ccl2	5’GGTCCCTGTCATGCTTCTGG 3’<br>3’CCTGCTGCTGGTGATCCTCT 5’	(Zammit et al., 2013)	NM_011333.3
Pparg	*	*	NM_001127330.2
CD36(FAT)	5’CGCAGCCTCCTTTCCACCTTTTGT3’<br>3’ TGGTTGTCTGGATTCTGGAGGGGT5’	*	NM_001159556.1
Fatp1	5’CGCTTTCTGCGTATCGTCTG3’<br>3’GATGCACGGGATCGTGTCT5’	*	NM_011977.4
Fabppm	5’GCGTCCCGAGCAGTGGAAGGA3’<br>3’GGCATACGATTGGCAGAGGCAGA5’	*	NM_010325.2
Lpl	5’TGGCGTAGCAGGAAGTCTGA3’<br>5’TGCCTCCATTGGGATAAATGTC3’	*	NM_008509.2
B2m	5’TTCTGGTGCTTGTCTCCAGTATGTTC3’<br>5’GCTTCCCATTCACTGA3’	*	NM_011149.2
B-actina (Actb)	5’AAATCTGGCACCACACCTTC3’<br>5’GGGGTGTTGAAGGTCTCAAA3’	(Coughlin et al., 2016)	NM_007393.5
Gapdh	5’TGTGTCCGTCGTGGATCTGA 3’<br>3’TTGCTGTTGAAGTCGCAGGAG 5’	(Gong et al., 2014)	NM_001289726.1
Ywhaz	5’ GAAAAGTTCTTGATCCCCAATGC 3’<br>5’ TGTGACTGGTCCACAATTCCTT 3’	*	NM_011740.3

*Gene specific primers were designed with primer-BLAST (http://www.ncbi.nlm.gov/tools/primer-blast).

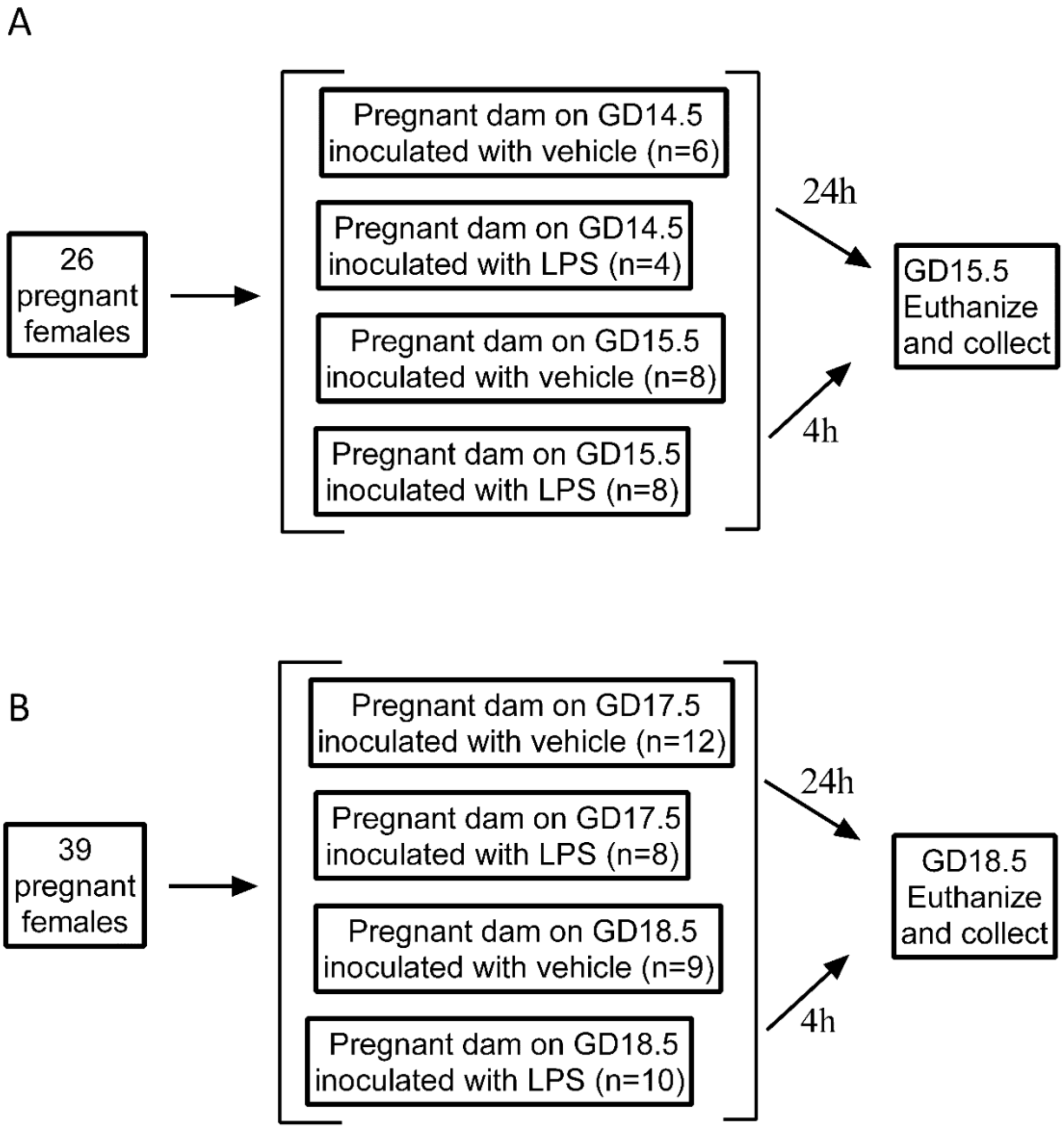

Supplementary figure 1: Study Design.

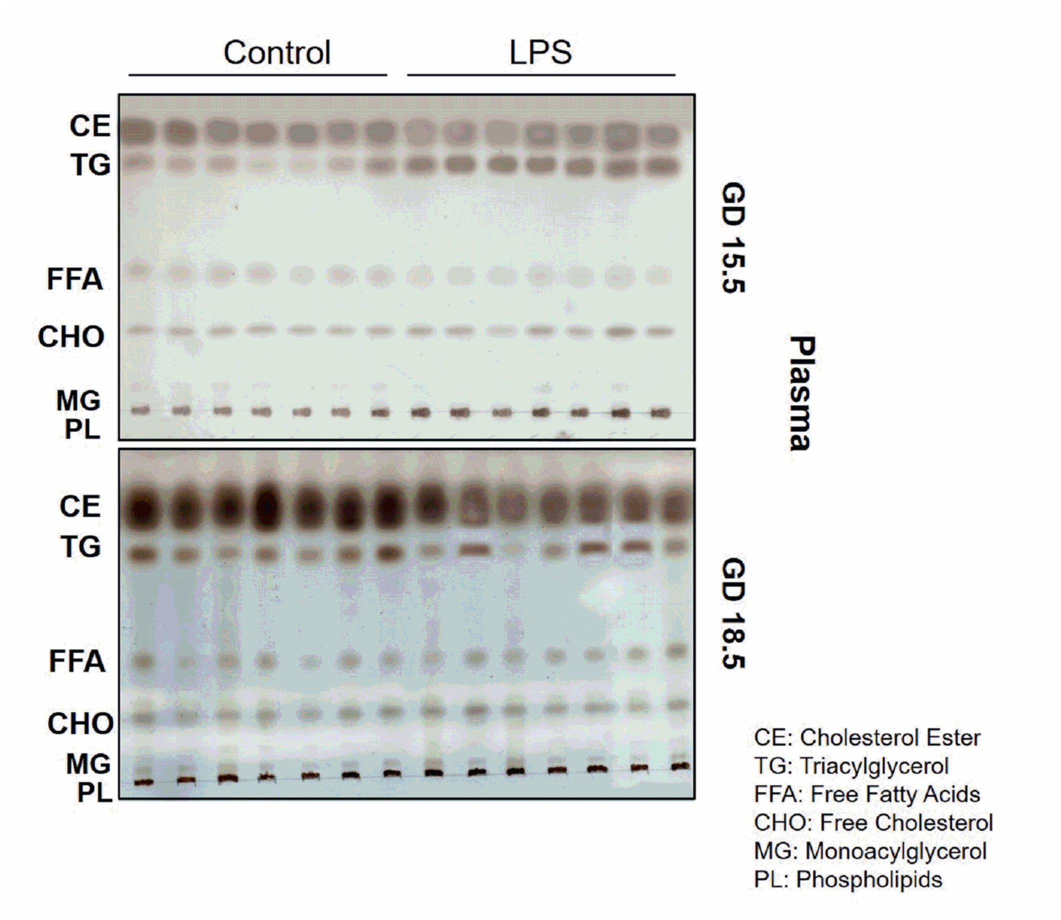

Supplementary figure 2: Representative image of TLC plate from maternal plasma at GD 15.5 and 18.5. 15.5/4h: n=6 (control group); n=5 (LPS group). CE: Cholesterol Ester; TG: Triacylglycerol; FFA: Free Fatty Acids; CHO: Free Cholesterol; MG: Monoacylglycerol; PL: Phospholipids.

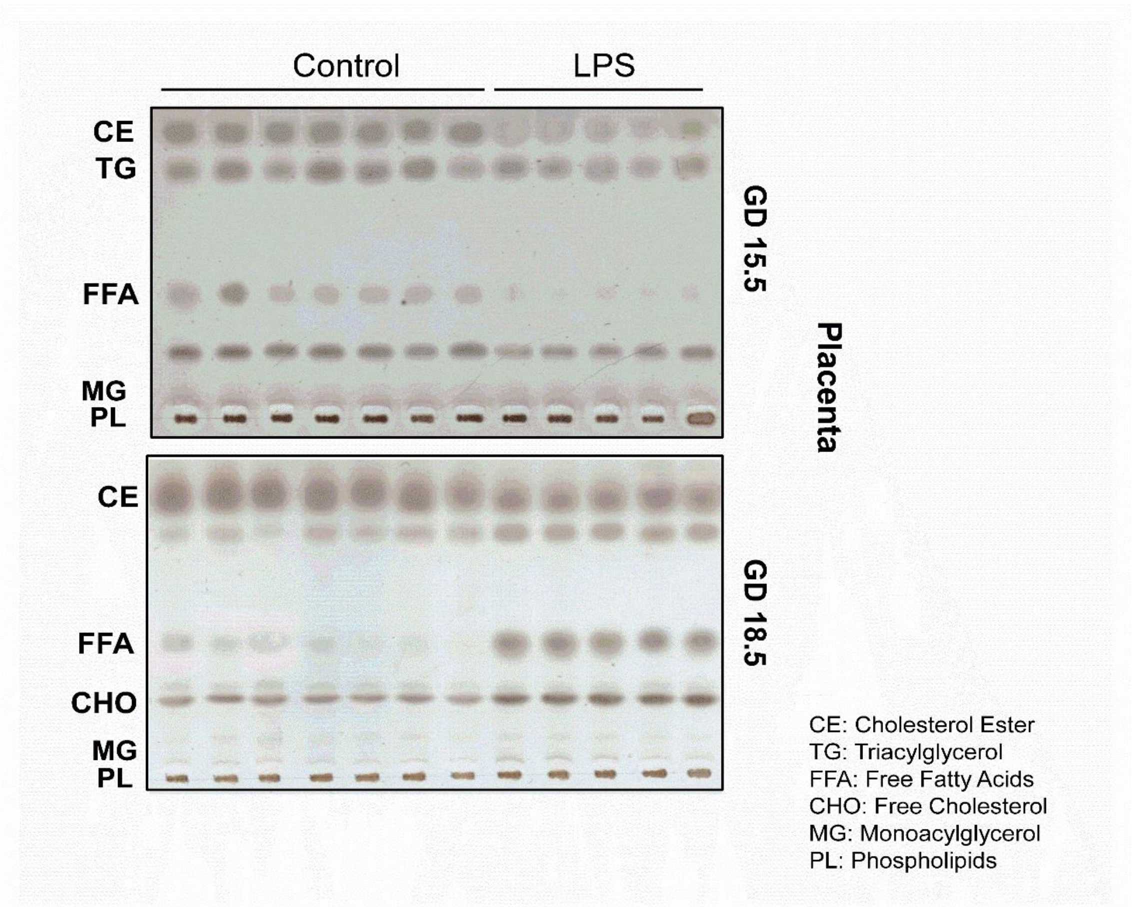

Supplementary figure 3: Representative image of TLC plate from placenta at GD 15.5 and 18.5. 15.5/4h: n=6 (control group); n=5 (LPS group); 18.5/4h: n=9 (control group); n=7 (LPS group). CE: Cholesterol Ester; TG: Triacylglycerol; FFA: Free Fatty Acids; CHO: Free Cholesterol; MG: Monoacylglycerol; PL: Phospholipids.

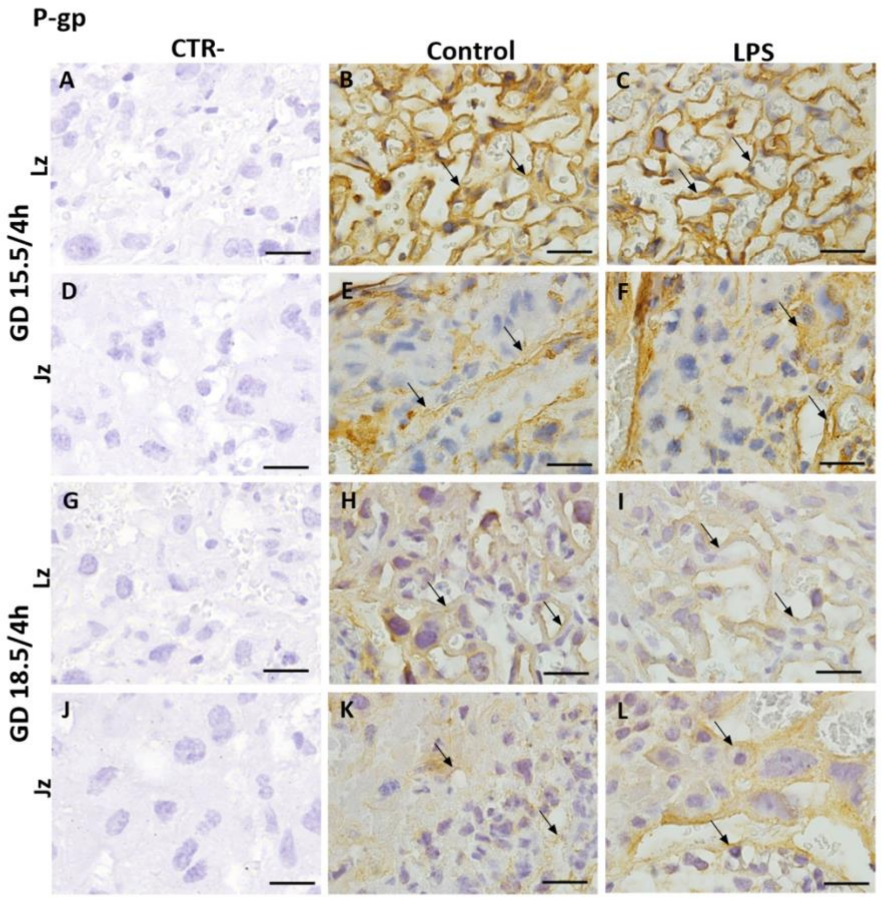

Supplementary figure 4: Representative photomicrographies of immunohistochemistry staining of P-gp in higher magnification 4 h after LPS insult at GD15.5 and 18.5 in the labyrinth (Lz) and junctional (Jz) zones of the placenta. n= 5/group. Scale bar =50 µm. Normal serum was incubated with negative control sections instead of the P-gp primary antibody.

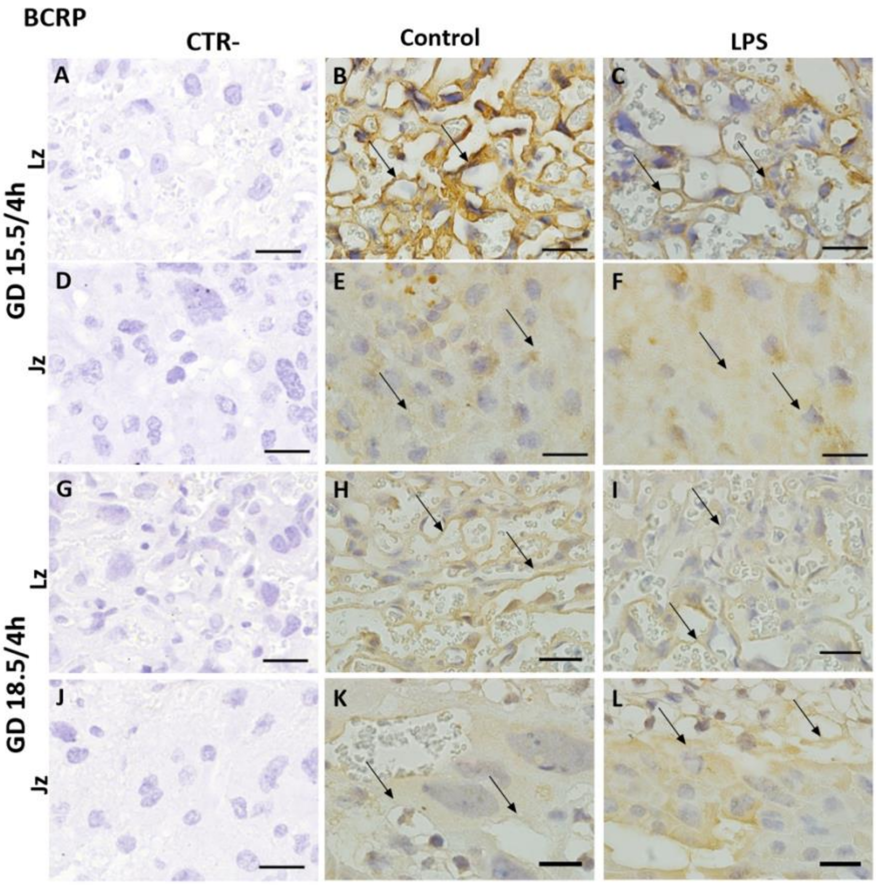

Supplementary figure 5: Representative photomicrographies of immunohistochemistry staining of Bcrp in higher magnification 4 h after LPS insult at GD15.5 and 18.5 in the labyrinth (Lz) and junctional (Jz) zones of the placenta. n= 5/group. Scale bar =50 µm. Normal serum was incubated with negative control sections instead of the Bcrp primary antibody.

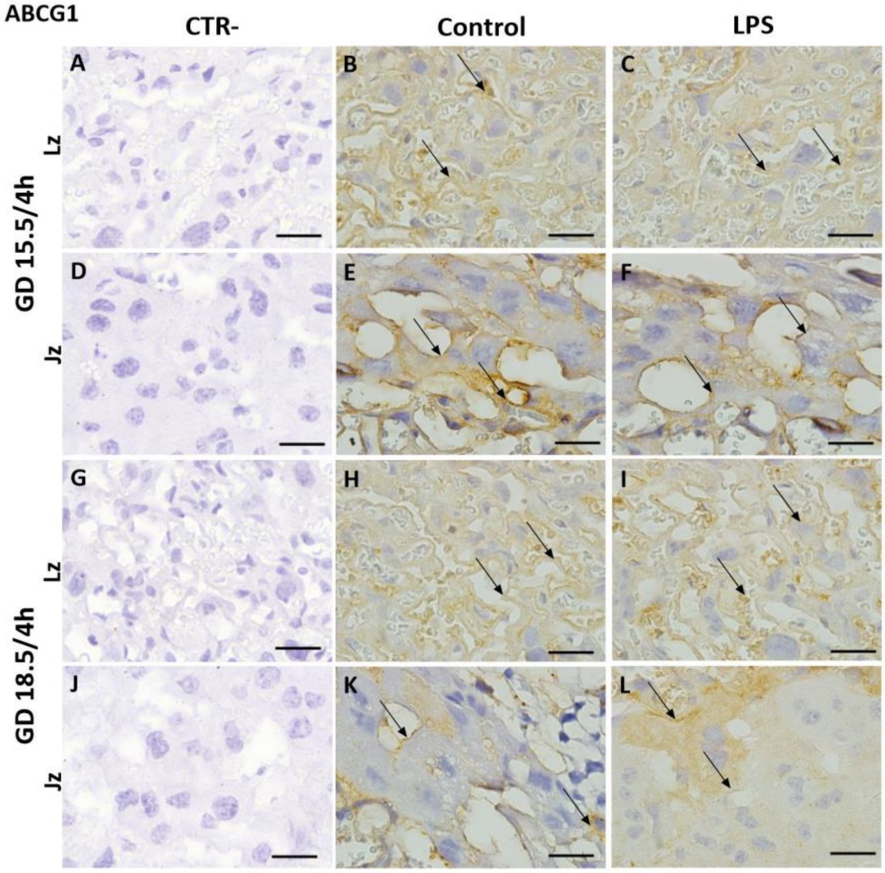

Supplementary figure 6: Representative photomicrographies of immunohistochemistry staining of Abcg1 in higher magnification 4 h after LPS insult at GD15.5 and 18.5 in the labyrinth (Lz) and junctional (Jz) zones of the placenta. n= 5/group. Scale bar =50 µm. Normal serum was incubated with negative control sections instead of the Abcb1 primary antibody.
